# Enhanced Recovery after Surgery in patients undergoing total joint arthroplasty: A retrospective study

**DOI:** 10.12669/pjms.39.3.7169

**Published:** 2023

**Authors:** Xizhen Zhao, Lingmin Chen, Feiri Huang, Zhihong Huang, Huijie Zhou

**Affiliations:** 1 Xizhen Zhao Department of Orthopedics, The Third Affiliated Hospital of Shanghai University (Wenzhou People’s Hospital), Wenzhou 325000, Zhejiang Province, P.R. China; 2 Lingmin Chen Department of Urology Surgery, The Third Affiliated Hospital of Shanghai University (Wenzhou People’s Hospital), Wenzhou 325000, Zhejiang Province, P.R. China; 3 Feiri Huang Department of Orthopedics, The Third Affiliated Hospital of Shanghai University (Wenzhou People’s Hospital), Wenzhou 325000, Zhejiang Province, P.R. China; 4 Zhihong Huang Department of Nursing, The Third Affiliated Hospital of Shanghai University (Wenzhou People’s Hospital), Wenzhou 325000, Zhejiang Province, P.R. China; 5 Huijie Zhou Department of Nursing, The Third Affiliated Hospital of Shanghai University (Wenzhou People’s Hospital), Wenzhou 325000, Zhejiang Province, P.R. China

**Keywords:** ERAS, Fast-recovery, Knee arthroplasty, Hip arthroplasty, Joint

## Abstract

**Objective:**

Enhanced Recovery after Surgery (ERAS) protocol has been developed and practiced for various surgical procedures to improve outcomes in the postoperative period. We hereby present our experience of ERAS for a large cohort of patients undergoing total joint arthroplasty (TJA).

**Methods:**

We implemented the ERAS program at The Third Affiliated Hospital of Shanghai University from January 2020 and retrospectively compared outcomes of patients undergoing total knee or hip arthroplasty before and after the implementation of the program. ERAS protocol consisted of the use of patient education, blood management, multimodal analgesia, antiemetics, shorter fasting time, no patient-controlled analgesia, early physical therapy, and reduced use of catheters and drains.

**Results:**

There were 94 patients in the study group (ERAS) and 113 patients in the control group (non-ERAS). We noted a statistically significant reduction in the incidence of postoperative nausea/vomiting, lowered pain scores, reduced length of hospital stay and better functional outcomes with both total knee and hip arthroplasties in our study cohort.

**Conclusion:**

ERAS protocol can bae effectively implemented for patients undergoing TJA. The use of ERAS leads to better postoperative outcomes and shortened hospital stay.

## INTRODUCTION

Patients with debilitating joint diseases make a significant portion of patients visiting an orthopedic clinic. Not surprisingly, total joint arthroplasty (TJA) has become one of the commoner surgical procedures in clinical practice. A large proportion of TJAs consists of either total hip arthroplasty (THA) or total knee arthroplasty (TKA).^1^ Recent data indicates that there would be an increase in the number of TJAs by approximately 85% by the end of the current decade.^2^ Considering the large number of TJAs performed globally, there should be an effort from all the surgical and nursing personnel to reduce perioperative patient morbidity while focusing on decreasing patient costs and improving post-surgical satisfaction.^3^ Data indicates that inadequate perioperative management with high rates of complications can increase the costs of THA and TKA by 22,775$ and 24,183$ respectively.^4^

The Enhanced Recovery After Surgery (ERAS) protocol was initially advocated by a Danish surgeon Kehlet H in 1997.^5^cardiopulmonary, infective and thromboembolic complications, cerebral dysfunction, nausea and gastrointestinal paralysis, fatigue and prolonged convalescence. The key pathogenic factor in postoperative morbidity, excluding failures of surgical and anaesthetic technique, is the surgical stress response with subsequent increased demands on organ function. These changes in organ function are thought to be mediated by trauma-induced endocrine metabolic changes and activation of several biological cascade systems (cytokines, complement, arachidonic acid metabolites, nitric oxide, free oxygen radicals, etc He promulgated the use of several evidence-based perioperative interventions to attenuate the physical and psychological trauma and stress experienced by the patient in the postoperative period to improve the recovery process. The ERAS aims to decrease the stress and trauma of the procedure by using less invasive surgical practices to reduce adverse events, shorten hospital stay, improve patient satisfaction, and aid in faster recovery.^6^ Since its initial application in gastrointestinal surgeries^7^, the ERAS has been promulgated in several other surgical procedures as well. Since The documentation of its use orthopedic procedures is not adequate thence we present our experience of ERAS for a large cohort of TJA patients.

## METHODS

A retrospective study with a control group was designed to assess the efficacy of the ERAS program on patients undergoing THA/TKA. The ERAS program was introduced in our institute in July 2021 and we included all patients undergoing THA/TKA in this program as the study group (July 2021 to December 2022). The control group consisted of patients undergoing THA/TKA before the introduction of ERAS (January 2020 to June 2021) we retrieved the institutional medical records of all patients undergoing THA/TKA from January 2020 to June 2021. The records were De-identified to maintain patient confidentiality. The data obtained after the intervention was compared to the data obtained retrospectively from past records.

### Ethical approval:

Institutional ethical committee approval was obtained before beginning the study (No. 2021-303, date: 2021-12-20). Informed written consent was obtained from all patients for the surgical procedures.

### Inclusion Criteria:

- Patients had no serious underlying medical comorbidities.
- Those undergoing THA/TKA for the first time.
- Who were mentally sound to understand the study plan and follow the instructions, and were cooperative to perform various exercises before and after the operation.
- Had support from family members
- Whose complete medical records were available and they completed regular follow-up up to three months after surgery.

### Exclusion Criteria:

We excluded patients with serious medical comorbidities, those with poor cognitive ability, on long-term anticoagulant therapy and those undergoing revision THA/TKA.

### ERAS and control program:

In the control group, the patients were included a few days before the surgery. Standard surgical protocols were followed. Surgery was done under general, spinal, or epidural anesthesia. Post-operative drainage was used in most patients. No standard pain control method was used and pain management was at the discretion of the treating physician. Progressive physiotherapy was carried out and patients were discharged to a rehabilitation center.

For the ERAS group, the surgery protocol involved initial interaction with the surgeon which was complemented by a joint replacement educational program. Patients were screened for iron deficiency anemia and treated accordingly. A detailed surgery information document was prepared and handed over to all patients. Patients were admitted a few days before surgery and the discharge date was predetermined. Preoperative fasting time was reduced. As a part of the blood-sparing strategy, tranexamic acid was given preoperatively as an intravenous agent and continued postoperatively in oral form. Preemptive analgesia in the form of buprenorphine transdermal patch 5mg was applied to the subclavian pectoralis major muscle in all patients. Patient-controlled analgesia (PCA) was omitted instead a fixed dose of multimodal pain regimen was used. Intra articular injection of a cocktail of tranexamic acid 1.2g, morphine 10mg, ropivacaine 75mg and epinephrine 0.5mg was also used after the surgery for postoperative pain and hemorrhage control.

The use of anti-emetics was also standardized. The use of drains was restricted and if used they were removed as soon as possible. Early walking was encouraged in the recovery room. Similarly, early intake of fluids was encouraged. Intravenous fluid therapy was withdrawn in 6-8 hours and the patient was returned to the ward. Oral antimicrobial therapy was then initiated. All patients were encouraged to do active self-rehabilitation with the aid of the physiotherapist in the initial stage. Discharge time was endorsed by the operating surgeon on the first postoperative day itself.

### Data and outcomes:

Pre-operatively, we collected information on patients’ demographics, history of diabetes, patient diagnosis, side of THA/TKA, type of anesthesia, pre-operative length of stay (LOS), fasting hours before surgery, and hemoglobin (Hb) and glucose levels before surgery. Outcome data consisted of days of catheterization post-surgery, use and duration of the wound drainage tube, time to oral intake post-surgery, the requirement of blood transfusion, Hb and glucose levels, post-operative nausea and vomiting (PONV), incidence of venous thromboembolism (VTE), pain scores, postoperative LOS, range of motion (ROM) (on 1^st^ and 3^rd^ postoperative days), Hospital for special surgery (HSS) knee rating scale, and Harris score recorded at one week, one month and three months.

### Statistical analysis:

SPSS 19.0 statistical software was used for data analysis. Continuous data were expressed as mean and standard deviation while ordinal data was represented by rates and percentages. The student’s t-test was used to compare continuous data while frequencies were compared using the Chi-square test or Fisher’s exact test as appropriate. P values of <0.05 were considered statistically significant.

## RESULTS

A total of 94 patients underwent THA/TKA under the ERAS program in the specified study period. The control group consisted of 113 patients who underwent THA/TKA before the introduction of ERAS. No patient was lost to follow-up during the study. The baseline details of included TKA and THA patients are presented in Table-I and Table-II respectively. For TKA, there was no statistically significant difference in gender and percentage of diabetics between the two groups. The study group had a significantly higher number of patients with bilateral knee osteoarthritis while the control group had a higher number of patients with left osteoarthritis.

**Table-I:** Baseline details of study and control group-TKA

Variable	Study group	Control group	p-value
Sample size	50	57	
Age	78± 7.4	77.5± 8.8	0.75
Male Gender	17 (34%)	22 (38.6%)	0.24
Diabetic	12 (24%)	13 (22.8%)	0.88
Diagnosis			<0.0001
B/L knee OA	26 (52%)	10 (17.5%)	
Right knee OA	22 (44%)	24 (42.1%)	
Left knee OA	2 (4%)	21 (36.8%)	
Trauma	0	2 (3.5%)	
TKA right/left	31/19	30/27	0.32
Anesthesia			0.0004
Spinal, epidural & nerve block	18 (36%)	38 (66.7%)	
Spinal & epidural	22 (44%)	7 (12.35)	
GA & nerve block	2 (4%)	5 (8.8%)	
GA only	8 (16%)	5 (8.8%)	
Epidural only	0	2 (3.5%)	
Preoperative hospital stay (days)	3.6± 1.73	4.42± 2.58	0.06
Fasting before surgery (solids) (hours)	5.76± 0.96	6.88± 1.89	0.0003
Fasting before surgery (liquids) (hours)	2± 0	2.77± 0.98	0.0001
Hb before surgery (g/L)	117.7± 19.19	122.88± 19.14	0.16
Blood glucose before surgery (mmol/L)	7.46± 1.92	7.26± 2.16	0.61

OA, osteoarthritis; B/L bilateral; Hb, hemoglobin.

**Table-II:** Baseline details of study and control group-THA.

Variable	Study group	Control group	p-value
Sample size	44	56	
Age (years)	79± 8.5	76.4± 10.8	0.19
Male gender	14 (31.8%)	20 (35.7%)	0.68
Diabetics	5 (11.4%)	11 (19.6%)	0.26
Diagnosis			0.01
Right femoral neck fracture	14 (31.8%)	27 (48.2%)	
Left femoral neck fracture	30 (68.2%)	21 (51.8%)	
Anesthesia			<0.0001
GA & nerve block	8 (18.2%)	43 (76.8%)	
Spinal & epidural	33 (73%)	9 (16.1%)	
Peripheral nerve block only	3 (6.8%)	0	
Epidural only	0	4 (7.1%)	
Preoperative hospital stay (days)	4.68± 4.67	4.77± 2.82	0.9
Fasting before surgery (solids) (hours)	5.73± 1.02	7.93± 1.41	<0.0001
Fasting before surgery (liquids) (hours)	2± 0	4± 1	<0.0001
Hb before surgery (g/L)	114.05± 20.32	120.2± 15.66	0.09
Blood glucose before surgery (mmol/L)	7.22± 1.52	7.63± 2.2	0.61

Hb, hemoglobin; GA, general anesthesia.

There was no difference in the side of TKA (right/left) between the two groups. Most patients in both groups underwent surgery under spinal and epidural anesthesia. However, peripheral nerve blocks were more frequently used in the control group. Ten patients in both groups underwent surgery under general anesthesia with or without peripheral nerve blocks. The preoperative LOS did not differ significantly between the two groups. The fasting time for both solids and liquids was significantly shorter in the study as compared to the control group. Pre-operative Hb and glucose levels did not differ significantly between the two groups.

Similarly, for THA, there was no difference in the age, gender, and percentage of diabetics between the two groups. Spinal and epidural anesthesia was more commonly used in the study group while GA was frequently used in the control group. Fasting time before surgery was significantly shorter in the study group. There was no difference in baseline Hb and glucose levels. Postoperative outcome data for TKA patients is presented in Table-III. Postoperative catheterization days were significantly reduced in the study as compared to the control group. However, the use of wound drainage was significantly lower in the study group.

**Table-III:** Outcome data-TKA.

Variable	Study group	Control group	P-value
Catheterization days	1.8± 1.11	2.82± 2.73	0.01
Wound drainage tube	9 (15.8%)	20 (40%)	0.004
Number of days of drainage tube	0.18± 0.60	0.98± 1.25	0.0001
Use of PCA	0	49 (98%)	<0.0001
PONV	3 (6%)	15 (26.3%)	<0.0001
Drinking clear fluids within 2 hours	50 (100%)	34 (59.6%)	<0.0001
Blood transfusion	8 (16%)	9 (15.8%)	0.97
Hb up to 3 days post-surgery (g/L)	98.54± 15.17	99.34± 15.32	0.78
Hb at discharge (g/L)	95.66± 13.26	98.22± 13.62	0.30
Blood glucose post surgery (mmol/L)	6.6± 1.65	6.59± 1.87	0.97
VTE	1 (2%)	6 (10.5%)	0.11
ROM POD 1 (degree)	59.4± 3.45	47.54± 2.52	0.0001
ROM POD 3 (degree)	96± 5.98	70.09± 3.47	0.0001
VAS 12 hours	2.36± 0.5	4.32± 0.72	0.0001
VAS 24 hours	1.43± 0.51	2.77± 0.53	0.0001
VAS 72 hours	0.86± 0.36	2.05± 0.49	0.0001
HSS score x 1 week	64.12± 2	61.4± 1.8	0.0001
HSS score x 1 month	86.58± 2.02	79.3± 2.84	0.0001
HSS score x 3 months	89.52± 1.52	87.02± 2	0.0001
Postoperative hospital stay (days)	7.56± 2.89	11.63± 7.23	0.003

ROM, Range of motion; POD, postoperative day; HSS, Hospital for special surgery knee rating scale; Hb, hemoglobin; VTE, venous thromboembolism; PCA, Patient controlled analgesia; PONV, postoperative nausea and vomiting; VAS, visual analog scale.

While the drains were removed in 2-3 days in all patients, the duration of the presence of a drainage tube was significantly shorter in the study as compared to the control group. All patients in the study group were able to drink clear fluids within two hours of surgery while the corresponding figure was only 59.6% in the control group. PONV was significantly reduced in the study group. Blood transfusion was needed in 16% of patients in the study group and 15.8% of patients in the control group with no statistically significant difference. VTE events were noted in one patient in the study group and six in the control group but the difference was not statistically significant. The postoperative LOS was significantly shorter in the study as compared to the control group. Patients in the study group had significantly better ROM on both the first and third postoperative days. The HSS score was also significantly higher in the study group at all three follow-up times (one week, one month, and three months).

Postoperative outcome data for THA patients are presented in Table-IV. Postoperative catheterization days were reduced in the study as compared to the control group, but the difference did not achieve statistical significance (p=0.05). While the use of drainage did not differ between the two groups, the time of postoperative drain was significantly shorter in the study group. All patients were able to drink clear fluids two hours post-surgery in the study group while only 5.4% of patients had fluids in the control group. There was no difference in the incidence of blood transfusion and VTE between the two groups while PONV was significantly reduced in the study group. Pain scores, Harris scores, and LOS were significantly better in the study group as compared to the control group.

**Table-IV:** Outcome data-THA

Variable	Study group	Control group	P-value
Catheterization days	2.18± 1.6	3.2± 3.17	0.05
Wound drainage tube	24 (54.5%)	19 (33.9%)	0.38
Number of days of drainage tube	0.8± 1.26	1.34± 1.29	0.03
Use of PCA	0	44 (100%)	<0.0001
PONV	2 (4.5%)	10 (17.9%)	0.04
Drinking clear fluids within 2 hours	44 (100%)	3 (5.4%)	<0.0001
Blood transfusion	11 (25%)	20 (35.7%)	0.97
Hb up to 3 days post-surgery (g/L)	94.77± 16.64	95.02± 15.95	0.93
Hb at discharge (g/L)	95.4± 13.9	93.27± 12.10	0.41
Blood glucose post surgery (mmol/L)	6.47± 1.29	6.73± 2.37	0.51
VTE	0	3 (5.4%)	0.11
VAS 12 hours	2.36± 0.69	4.3± 0.71	0.0001
VAS 24 hours	1.75± 0.61	2.82± 0.58	0.0001
VAS 72 h	1.09± 0.29	2.41± 0.50	0.0001
Harris score x 3 days	54.64± 2.64	43.43± 2.16	0.0001
Harris score x 1 week	63.86± 2.39	51.54± 2.34	0.0001
Harris score x 1 month	76.48± 2.89	70.88± 3.57	0.0001
Harris score x 3 month	87.2± 2.18	81.39± 2.09	0.0001
Harris score x 6 month	91.23± 1.31	87.48± 1.50	0.0001
Postoperative hospital stay (days)	5.73± 1.02	12.11± 8.85	0.0001

ROM, Range of motion; POD, postoperative day; Hb, hemoglobin; VTE, venous thromboembolism; PCA, Patient controlled analgesia; PONV, postoperative nausea and vomiting; VAS, visual analog scale.

## DISCUSSION

In the past decade, there has been a spurt in the use of ERAS protocols across several different surgical specialties with the sole purpose of boosting patient recovery and optimizing surgical outcomes with a minimal increase in healthcare expenditure. The ERAS set of guidelines was initially developed to streamline perioperative care so that it could improve patient satisfaction and reduce the rate of complications. Indeed, there have been numerous reports and randomized controlled trials (RCTs) supporting the use of ERAS protocols in non-TJA procedures.^7^-^10^

Ni et al^7^ in a meta-analysis of 13 RCTs have shown that the ERAS program is better and safer for patients undergoing laparoscopic colorectal surgery with reduced LOS, time to first flatus, time to first defecation and lower rate of complications. Similarly, another meta-analysis including five RCTs has noted ERAS to be effective in reducing LOS, complications, and time to flatus in patients undergoing liver surgery as well.^10^ The success of ERAS has also been seen in cancer patients with meta-analyses studies reporting significantly better outcomes in patients with breast, gastric and gynecological cancers.^8^,^9^,^11^ Such success of the program also encouraged the use of ERAS in various orthopedic procedures. Recently, Pennington et al^12^and there has been drive from providers and payors alike to decrease inpatient stays. One strategy currently being explored is the use of Enhanced Recovery After Surgery (ERAS in a systematic review and meta-analysis of 34 studies have demonstrated that ERAS reduces hospitalization time, pain scores, and complication rates in patients undergoing spine surgeries. Jiang et al^13^ in a pooled analysis of 57 studies have shown that the ERAS protocol reduces LOS, pain, healthcare expenditure, and complications while improving function in patients undergoing surgery for hip fracture. Since TJA is one of the most commonly performed elective orthopedic procedures, the program must be replicated in such patients as well.

In our experience of 207 cases, we too noted better outcomes with the implementation of the ERAS protocol in our healthcare setup. To be precise, we noted a reduced incidence of PONV, improved pain scores, reduced hospitalization time, and better functional outcomes with both THA and TKA in our study cohort. Similar outcomes have been reported by previous studies on TJA. Picart et al^14^ in a study of 551 patients undergoing TKA noted that ERAS was effective in reducing the LOS and pain scores but with no difference in infection rates, readmission, or surgical revision. Berg et al^15^ in a large before-after study of 14,148 TJA noted that ERAS effectively reduced LOS but with no difference in the risk of readmissions or adverse events.

A similar statistically significant reduction in LOS was also noted by Christelis et al^16^ after the implementation of ERAS in a cohort of 412 patients undergoing THA or TKA. Zhu et al^17^ in a pooled analysis of 10 studies on TJA have shown that ERAS reduces LOS and complications with no change in the incidence of readmissions. LOS is an important patient-related outcome that is consistently reduced by ERAS in TJA as well as for other surgical specialties. Reducing hospitalization time not only reduces healthcare expenditure but also improves patient satisfaction and improves functional outcomes in patients undergoing TJA.^18^

While the evidence supports the implementation of ERAS for TJA procedures, it is important to note that there are several elements to the ERAS with different healthcare systems adopting different components based on individual feasibility.^19^ An expert group of Chinese TJA surgeons has recommended the use of better patient education, nutrition supplementation, optimization of anesthesia, reduced fasting time, use of carbohydrates in the perioperative period, early resumption of feeding, restrictive fluid therapy, minimally invasive surgery, blood and anemia management, prevention of VTE, multimodal analgesia, management of PONV, reduced use of catheter and drains, sleep management, early functional exercises and routine follow-up post-discharge for the ERAS program in joint arthroplasty patients.

Indeed, not all of these ERAS components were implemented in our healthcare setup with few elements missed out like use of carbohydrates in the perioperative period, minimally invasive surgery, and sleep management; but we managed to execute most of the major elements thereby improving patient outcomes. Chen et al^19^ have shown that ERAS protocols have not been consistent for TJA patients but most have used multimodal analgesia, antiemetics, no PCA, early physical therapy, and reduced use of catheters and drains which is similar to our study. The authors noted that despite a different combination of ERAS elements there was little change in outcomes and further studies are required to isolate individual components and their combinations to completely comprehend their efficacy and benefits.

### Limitations:

Our study was retrospective in design with a historical control group which made it prone to bias. Another limitation was that sample size of the study was not large. Thirdly, we were unable to analyze data on complication rates, infection rates, and readmission rates due to the lack of availability of data. Lastly, we were able to assess functional outcomes only for the first six months. Long-term data would be provided better evidence.

Nevertheless, the current study supplements evidence from medical literature that ERAS protocol can lead to better patient outcomes in patients undergoing TJA. These finding assume clinical significance as these easy to implement protocols can be adopted in any healthcare setup resulting in improved patient satisfaction after TJA surgery.

## CONCLUSION

ERAS protocol can be effectively implemented for patients undergoing TJA. ERAS led to a significant reduction in the incidence of PONV, reduced pain scores, and better functional scores in both THA and TKA.

### Authors’ contributions:

**XZ and LC:** Conceived and designed the study.

**FH, ZH and HZ**: Collected the data and performed the analysis.

**XZ and LC**: Were involved in the writing of the manuscript and are responsible for the integrity of the study.

All authors have read and approved the final manuscript.
